# N-doped carbon anchored CoS_2_/MoS_2_ nanosheets as efficient electrocatalysts for overall water splitting

**DOI:** 10.1007/s12200-022-00034-3

**Published:** 2022-07-18

**Authors:** Xingwei Zhou, Wei Zhang, Zunhao Zhang, Zizhun Wang, Xu Zou, Dabing Li, Weitao Zheng

**Affiliations:** 1 Key Laboratory of Automobile Materials MOE, School of Materials Science and Engineering, Electron Microscopy Center, and International Center of Future Science, Jilin Provincial International Cooperation Key Laboratory of High-Efficiency Clean Energy Materials, Jilin University, Changchun, 130012 China; 2 Wuhan National Laboratory for Optoelectronics, Huazhong University of Science and Technology, Wuhan, 430074 China; 3 State Key Laboratory of Luminescence and Applications, Changchun Institute of Optics, Fine Mechanics and Physics, Chinese Academy of Sciences, Changchun, 130033 China

**Keywords:** Oxygen evolution reaction, Hydrogen evolution reaction, Bifunctional electrocatalyst, Overall water splitting

## Abstract

The oriented two-dimensional porous nitrogen-doped carbon embedded with CoS_2_ and MoS_2_ nanosheets is a highly efficient bifunctional electrocatalyst. The hierarchical structure ensures fast mass transfer capacity in improving the electrocatalytic activity. And the greatly increased specific surface area is beneficial to expose more electrocatalytically active atoms. For oxygen evolution reaction (OER) and hydrogen evolution reaction (HER) tests in 1 mol/L KOH solution, only 194 and 140 mV overpotential are required to achieve a current density of 10 mA/cm^2^, respectively. Our research provides an effective strategy for synergizing the individual components in nanostructures for a wide range of electrocatalytic reactions.

**Graphical Abstract:**

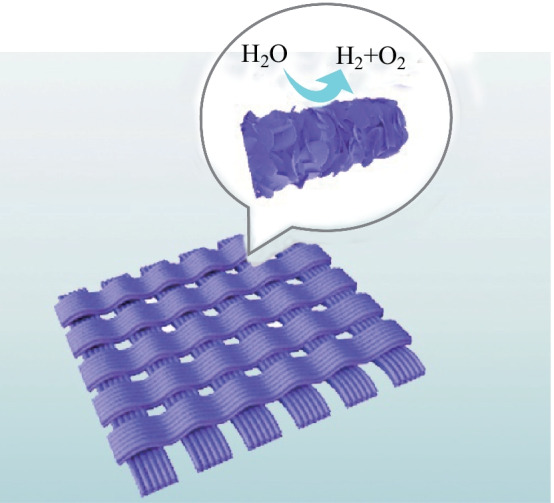

**Supplementary Information:**

The online version contains supplementary material available at 10.1007/s12200-022-00034-3.

## Introduction

Electrochemical water splitting ensures green and safe conversion of electrical energy to chemical energy [1–4]. However, water splitting is a thermodynamically uphill process and thus needs to be modulated by efficient hydrogen and oxygen evolution catalysts simultaneously to lower the reaction energy barrier. Noble metal-based electrocatalysts have been widely explored by reducing the activation energy barrier of the reaction and improving the energy conversion efficiency [5]. However, limited resources and expensiveness of these precious metals, severely hamper their large-scale applications. Transition metals and their compounds have attracted much attention, as they are abundant on earth, and offer excellent electrochemical properties [6–9]. A large number of such noble-metal-free materials have been synthesized but are electrocatalytically active for hydrogen evolution reaction (HER) or oxygen evolution reaction (OER) only. In contrast, bifunctional electrocatalysts that are effective for both HER and OER are more advantageous for overall water splitting, but are less common. A bifunctional electrocatalyst can be more easily integrated into a single water splitting device, and the HER and OER performances of the electrocatalyst can be optimized simultaneously.

It is well known that the number of active sites is critical to the overall activity of electrocatalysts. These active sites usually appear at the surfaces, including steps, kinks and edges of nanoscale structures [10]. In recent years, molybdenum disulfide (MoS_2_) has received extensive attention. Theoretical studies have shown that the MoS_2_ edge atoms exhibit excellent catalytic activity, while the basal plane atoms are chemically inert. One approach is to activate the basal plane of inert atoms and reduce the band gap by doping transition metal atoms (e.g., Ni, Co, Fe, Zn, and Ru) and non-metal atoms (e.g., C, N, O, P, and Se), thus improving their electrocatalytic properties [11, 12]. The exotic atoms can produce a local electron density at the host atoms, which can modify the energy barrier of the reaction by breaking the periodicity of MoS_2_. Another way to increase the quantity of active sites of MoS_2_ is to increase the density of the edge atoms by constructing specific nanostructures [13, 14]. Besides, the catalytic activity of the inert atoms in the 2H-MoS_2_ basal plane can be enhanced by coupling with the conducting substrate (e.g., carbon cloth, nickel foam) [15].

Metal organic framework (MOF) is gradually becoming a new class of crystalline materials, which has attracted much attention due to its porosity, high specific surface and controllable properties [16–19]. These advantages ensure a high mass transfer capacity and a wealth of active sites, which are beneificial to the improvement of electrocatalytic activity. Besides, constructing electrocatalysts with rich hierarchical structures helps to improve the overall activity of electrocatalysts [20].

In this work, a bifunctional electrocatalyst MoS_2_/CoS_2_/carbon cloth (MOS/COS/CC) was prepared by embedding both CoS_2_ and MoS_2_ nanosheets in the oriented 2D porous N-doped carbon nanosheets. The porous structure of N-doped carbon nanosheets embedded with CoS_2_ nanosheets has positive effect on electrocatalytic activity. The introduction of MoS_2_ nanosheets greatly increases the specific surface area of the material, which is beneficial to the exposure of electrocatalytic active sites. Compared with use of powder electrocatalysts, employment of carbon cloth as the substrate greatly improves the conductivity and mechanical flexibility of the electrode. The interaction of the porous N-doped carbon nanosheets embedded with CoS_2_ and MoS_2_ nanosheets leads to the excellent performance of the bifunctional electrocatalysts. Testing the OER and HER performances of MOS/COS/CC in 1 mol/L KOH solution requires only an overpotential of 194 and 140 mV, respectively, to reach a current density of 10 mA/cm^2^. Our study provides an effective strategy for the construction of nanostructured bifunctional electrocatalysts.

## Experiment

### Chemicals and reagents

Cobalt nitrate hexahydrate (Co(NO_3_)_2_·6H_2_O), 2-methylimidazole (C_4_H_6_N_2_), platinum on carbon (20% Pt/C) and ruthenium (IV) oxide (RuO_2_) were purchased from Aladdin Chemistry Co., Ltd. Ethanol (CH_3_CH_2_OH), thiourea (CH_4_N_2_S), sodium molybdenum oxide (MoNa_2_O_4_) and hydroxyacetone (C_3_H_6_O_2_) were purchased from Sinopharm Chemical Reagent Co., Ltd. Carbon cloth was purchased from CeTech Co., Ltd. Highly purified water (> 18 MΩ⋅cm resistivity) was obtained from a PALL PURELAB Plus system.

### Synthesis of MOS/COS/CC

**Fig. 1:**
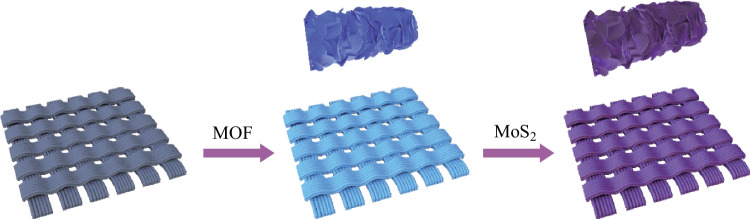
Schematic illustration of MOS/COS/CC electrocatalyst synthesis process

The synthesis process of the MOS/COS/CC electrocatalyst is represented in Fig. 1. First, the synthesis of MOF-CC was carried out using a solution route. Co(NO_3_)_2_· 6H_2_O (582 mg) and C_4_H_6_N_2_ (1.3 g) were dissolved in 25 mL of deionized water and recorded as A and B solutions, respectively. Then, after magnetic stirring for 0.5 h, solution A was quickly poured into solution B. The carbon cloth was placed in the obtained mixed solution and kept at room temperature for 4 h to obtain MOF-CC. Then, the MOF-CC was annealed in a tube furnace at 750 °C for 2 h. The heating rate was set as 5 °C/min. In the next step, CH_4_N_2_S (609 mg) was mixed with 30 mL of ionized water and stirred for 10 min. MoNa_2_O_4_ (483.9 mg) was added to the above solution and stirred for 30 min. The annealed sample was put into the mixed solution that was then placed in a reactor. The reactor was heated to 200 °C and maintained at that temperature for 2, 4, 6, and 8 h respectively to obtain four different samples. After hydrothermal reaction, MoS_2_ nanosheets were deposited on MOF-CC. A bifunctional electrocatalyst containing 2D porous N-doped nanosheets embedded with CoS_2_ and MoS_2_ nanosheets (MOS/COS/CC) was thus prepared.

### Electrochemical measurements

In the OER and HER tests, self-supporting catalysts were used as working electrodes. A graphite rod and saturated calomel (saturated KCl solution) electrodes were used as counter and reference electrodes, respectively. All potentials were converted to reversible hydrogen potentials. The Tafel slope was calculated by

$$\eta = a + b\log \left| j \right|,$$

where *η* is the overvoltage, *b* is the Tafel slope and *j* is the current density. The catalyst ink (5 mg/mL) was prepared by dispersing 5 mg of the catalyst into 1 mL of solution containing 0.73 mL of ultrapure water, 0.25 mL of absolute ethanol and 10 μL of Nafion (5%), followed by ultrasonication treatment for 0.5 h. Then 10 μL of the catalyst ink was deposited on the glassy carbon electrode (GCE) with loading of 0.25 mg/cm^2^. Linear sweep voltammetry (LSV)tests of OER and HER were performed at a scan rate of 5 mV/s in a solution of 1.0 mol/L KOH at room temperature, and the compensation potential was corrected by electrochemical impedance spectroscopy.

### Characterization

X-ray diffraction (XRD) analysis was conducted using a Bruker D8 with Cu Kα irradiation. Scanning electron microscopy (SEM, Hitachi SU8010) was employed for morphology analysis. Transmission electron microscopic (TEM) and high-resolution TEM (HRTEM) images were recorded using an electron microscope (JEM-2100F). The energy dispersive X-ray spectroscopy (EDS) was performed in scanning transmission electron microscopy (STEM). Surface chemical states of the catalysts were detected by using an X-ray photoelectron spectroscope (XPS, ESCALAB-250).

## Results and discussion

**Fig. 2:**
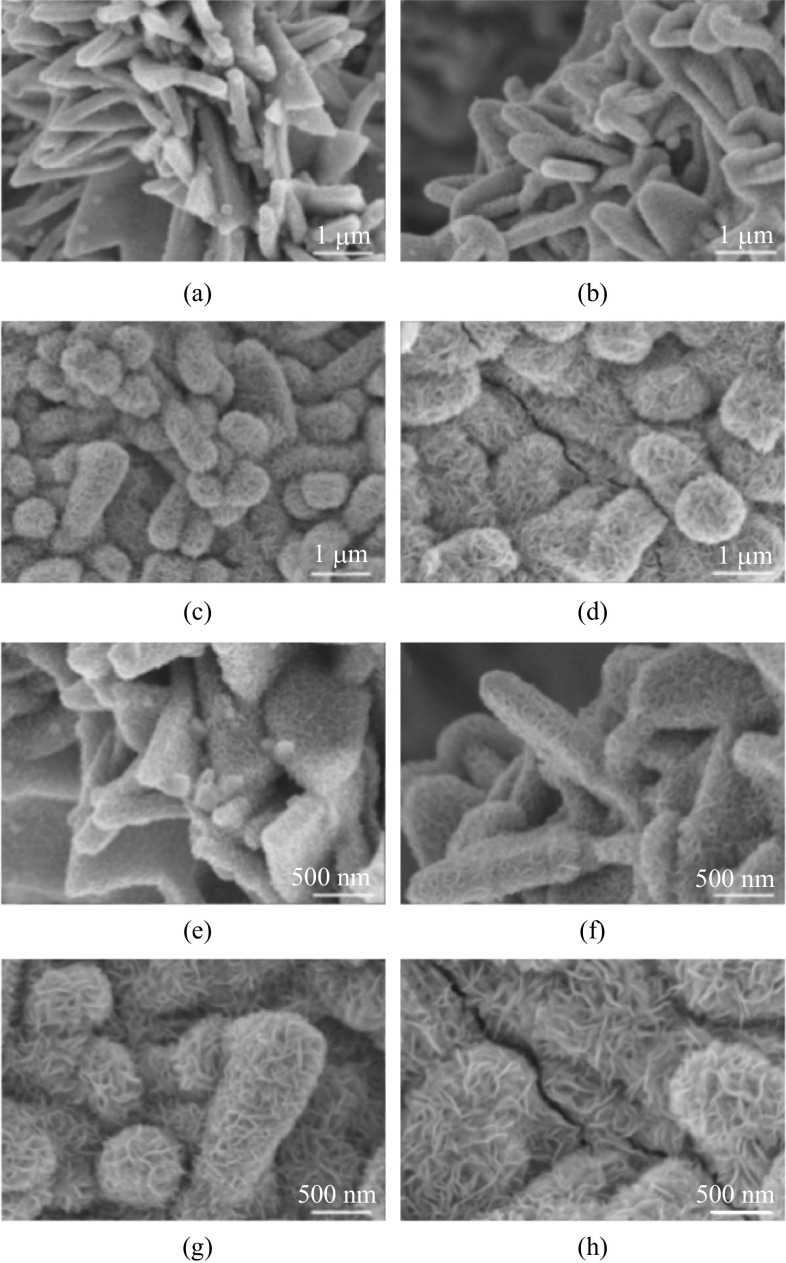
SEM images of MOS/COS/CC electrocatalysts with different synthesis time. **a**, **e** 2 h, **b**, **f** 4 h, **c**, **g** 6 h, and **d**, **h** 8 h

The heterostructure of MoS_2_ and CoS_2_ can be adjusted by controlling the growth time of MoS_2_, which has a significant effect on the performance of the electrocatalysts. Electrocatalysts with growth times of 2, 4, 6, and 8 h were investigated. These samples were recorded as MOS/COS/CC-2 h, MOS/COS/CC-4 h, MOS/COS/CC-6 h and MOS/COS/CC-8 h, respectively. As shown in Fig. 2, the number of MoS_2_ nanosheets deposited on the substrate increased with the growth time. As can be seen from Fig. 2a and e, the electrocatalysts with a deposition time of 2 h have a relatively small amount of MoS_2_ nanosheets growing on the substrate. Due to the low surface area, the active sites in MOS/COS/CC-2 h are less exposed. This did not support improvement of the catalytic activity of the electrocatalyst. Figure 2d and h show that the leaf-like nitrogen-doped nanosheets had been covered up and the cracks had appeared in the electrocatalyst due to the long growth time, which had a fatal influence on the performance and stability of the electrocatalyst. For Fig. 2f and g, the MoS_2_ nanosheets with a deposition time of 4 h and 6 h were the most impressive. The specific surface areas and number of active sites of MoS_2_ nanosheets were much larger than other samples.

**Fig. 3:**
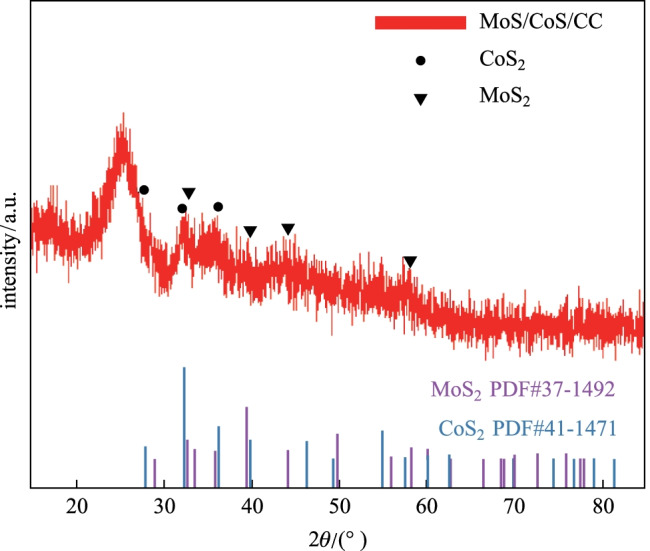
XRD patterns of MoS/CoS/CC electrocatalysts

**Fig. 4:**
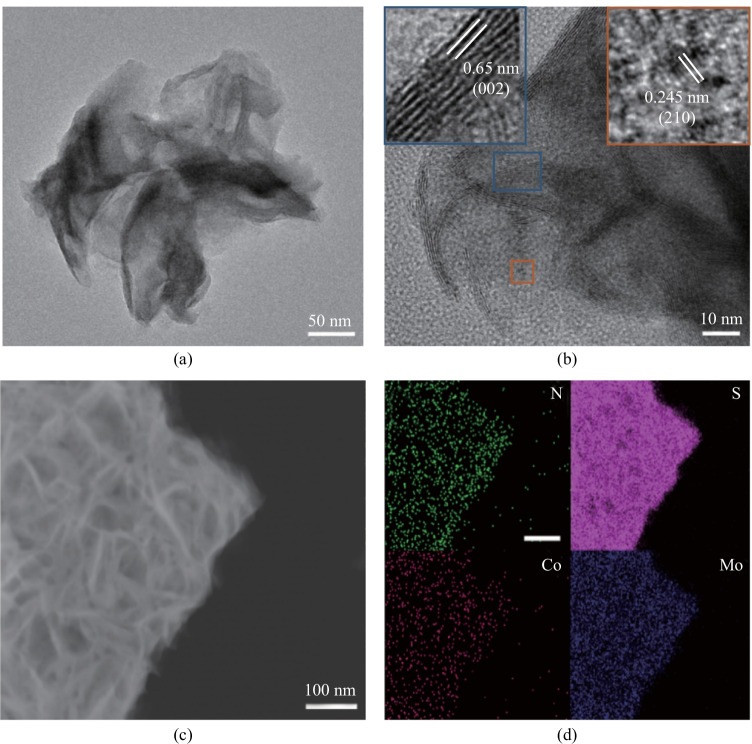
**a** and **b** TEM images of the MOS/COS/CC-6 h electrocatalyst. Insets are the local HRTEM images. **c** and **d** STEM-EDS elemental maps of N, S, Co and Mo of MOS/COS/CC-6 h

Figure 3 shows the XRD pattern of the MOS/COS/CC electrocatalyst. The XRD patterns of MOS/COS/CC electrocatalysts show diffraction peaks at 32.6° (100), 39.8° (103) and 58.3° (110), which are in good agreement with those of MoS_2_ in hexagonal 2H phase (PDF#37-1492). The diffraction angles of 27.8°, 32.3° and 36.2° correspond to the (111), (200) and (210) planes of CoS_2_ (PDF#41-1471), respectively. Figure 4a and b show that the interplanar spacing of the layered structure is 0.65 nm, which is consistent with the (002) plane of MoS_2_ with hexagonal 2H phase. In addition, the measured interplanar spacing of 0.245 nm corresponds to the (210) plane of CoS_2_. The results confirmed that MoS_2_ and CoS_2_ coexisted in the hybrid structure [21]. Figure 4c and d show the presence and uniform distribution of Mo, S, Co and N in the MOS/COS/CC electrocatalyst.

**Fig. 5:**
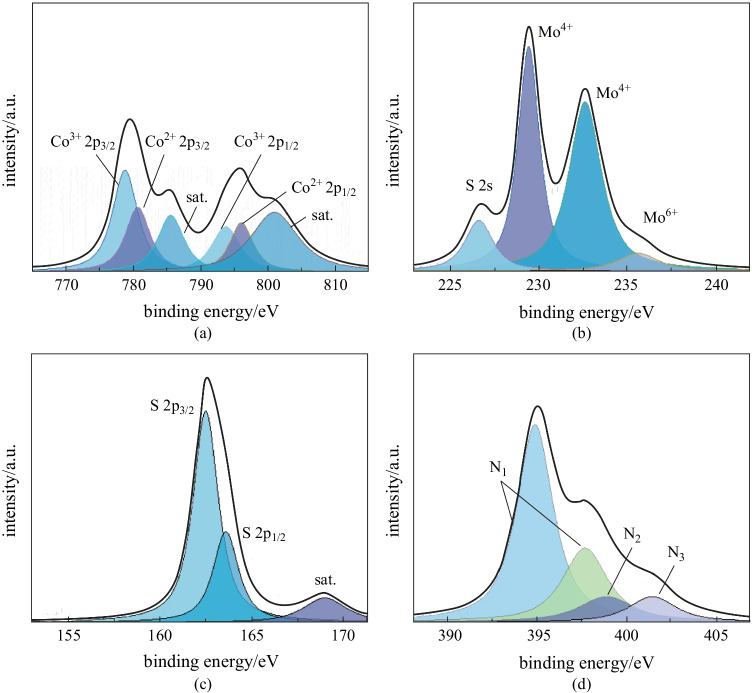
XPS spectra of MOS/CoS/CC-6 h electrocatalyst. **a** Co, **b** Mo, **c** S, **d** N

The chemical composition and valence of elements in MOS/COS/CC-6 h electrocatalyst were studied by XPS. From the XPS spectra of Co in Fig. 5a, it can be seen that the characteristic peaks of Co 2p orbit are deconvoluted into two pairs of spin orbital peaks. The characteristic peaks at 793.4 and 778.2 eV correspond to Co 2p_1/2_ and Co 2p_3/2_ from Co^3+^, while the characteristic peaks at 797.5 and 781.4 eV correspond to Co 2p_1/2_ and Co 2p_3/2_ from Co^2+^. Two characteristic peaks of Mo 3d were observed at 229.5 and 232.7 eV, corresponding to Mo 3d_5/2_ and Mo 3d_3/2_ respectively (Fig. 5b), indicating that Mo was in the valence state of +4. The small peak detected at 226.7 eV was attributed to S 2 s orbit. In addition, the S 2 s spectra could be deconvoluted into two characteristic peaks with binding energies of 226.9 and 227.5 eV, corresponding to the binding energies of S atoms in MoS_2_ and CoS_2_, respectively. The characteristic peaks of 162.5 and 163.6 eV binding energies in the spectra of S elements belonged to S 2p_3/2_ and S 2p_1/2_ respectively (Fig. 5c). In the corresponding N 1 s spectrum (Fig. 5d), two characteristic peaks at 395.02 and 397.82 eV were attributed to the Mo–N bond (N_1_), while the presence of pyridine N (N_2_, 399.03 eV) and graphite N (N_3_, 401.57 eV) indicated the successful binding of nitrogen atoms to the carbon substrate [22]. Compared with MOS/COS/CC-4 h and MOS/COS/CC-8 h, the content of Co element in MOS/COS/CC-6 h is in the middle, indicating that with the increase of MoS_2_ growth time, the Co element on the material surface gradually decreases (Additional file 1: Fig. S1). In addition, it can be seen from Additional file 1: Fig. S2 that, in the XPS spectrum of the MOS-COS-CC-6 h electrocatalyst, the binding energies of Mo 3d and 2 s shifted compared with those for the Mo element in the MoS_2_ electrocatalyst, indicating the interaction between MoS_2_ and CoS_2_ in the MOS-COS-CC electrocatalyst.

**Fig. 6:**
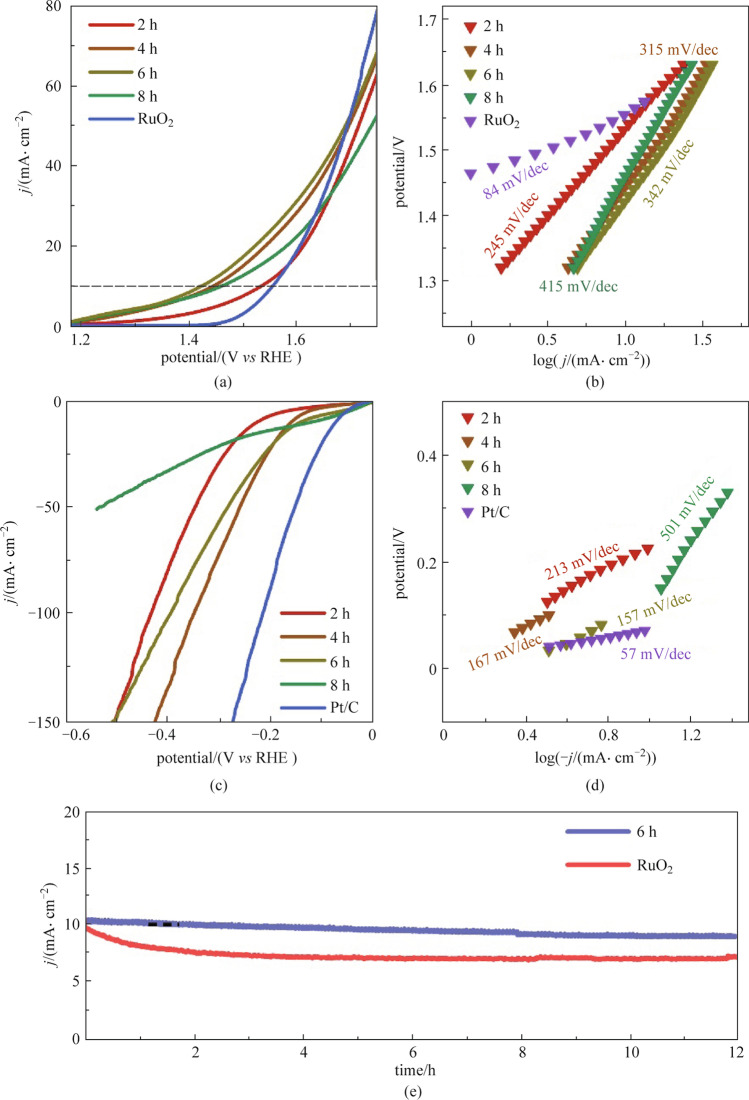
Electrochemical testing of RuO_2_ and MOS/COS/CC electrocatalysts with different synthesis time. **a** LSV curves of OER. **b** Tafel curves of OER. **c** LSV curves of HER. **d** Tafel curves of HER. **e** Stability test of MOS/COS/CC-6 h and RuO_2_ electrocatalysts

The OER properties of MOS/COS/CC with different growth time and commercial RuO_2_ electrocatalysts were tested in 1.0 mol/L KOH. Compared with other electrocatalysts, MOS/COS/CC-6 h only needs an overpotential of 197 mV for a current density of 10 mA/cm^2^ (Fig. 6a). The overpotential is significantly lower than that of commercial RuO_2_ and other electrocatalysts with different growth times. In addition, the MOS/COS/CC-6 h electrocatalyst had the lowest Tafel slope (64 mV/dec, Fig. 6b), which indicates that it has excellent chemical kinetics for electrocatalytic reaction. Concurrently, we also studied the HER properties of MOS/COS/CC with different growth time and commercial Pt/C electrocatalyst in 1.0 mol/L KOH (Fig. 6c). It can be seen that MOS/COS/CC-6 h and MOS/COS/CC-8 h only required overpotentials of 140 and 125 mV, respectively, to achieve a current density of 10 mA/cm^2^. However, the current density of MOS/COS/CC-8 h electrocatalyst decreased greatly at high potential. The reason is that the excessive deposition of MoS_2_ nanosheets, which is not conducive to the exposure of active sites and the cracking of the electrocatalyst surface, thus reducing the long-term stability of the electrocatalyst. The catalytic HER kinetics can be estimated from the Tafel diagram fitted by the LSV linear curve. As shown in Fig. 6d, the Tafel slope of MOS/COS/CC-6 h is 157 mV/dec, which is smaller than that of MOS/COS/CC-2 h (213 mV/dec) and MOS/COS/CC-8 h (501 mV/dec). This result demonstrates the faster HER catalytic kinetics of MOS/COS/CC-6 h. Furthermore, our material still exhibited relatively excellent catalytic activity in comparison with other bifunctional catalysts (in Table 1).

**Table 1:** Comparison of the electrocatalytic performance of MOS/COS/CC with recently reported bifunctional electrocatalysts for water splitting

Catalyst	Water electrolysis test	Current density (*j*)/(mA⋅cm^−2^)	Overpotential at the corresponding *j*/mV	Reference
MOS/COS/CC	HER^a^	10	140	This work
	OER^b^	10	194	
Co_5_Fe_5_-C	HER	10	165	[23]
	OER	10	245	
N-CoSe_2_@CP	HER	10	106	[24]
	OER	10	237	
ZnCo_2_S_4_/CoZn_13_	HER	10	160	[25]
	OER	50	274	
Fe-Co-Ni-S_*x*_/NF	HER	10	188	[26]
	OER	10	280	
MoS_2_-AB/NF	HER	10	77	[27]
	OER	10	248	
P-NiSe_2_@N-CNTs/NC	HER	10	95	[28]
	OER	10	206	
10:MoCo-VS_2_	HER	10	63	[29]
	OER	10	248	

^a^HER stands for hydrogen evolution reaction

^b^OER stands for oxygen evolution reaction

Besides high catalytic activity, MOS/COS/CC-6 h could also achieve good catalytic stability. As shown in Fig. 6e, the current density decreased by ~ 90% after 12 h reaction, while that of commercial RuO_2_ electrocatalyst decreased by 30%. This result indicates that MOS/COS/CC-6 h maintains high structural stability during the test.

## Conclusions

In conclusion, we have developed a simple and convenient method for constructing hierarchical structure. A bifunctional electrocatalyst with 2D porous N-doped nanosheets embedded with CoS_2_ and MoS_2_ nanosheets was synthesized by a hydrothermal method. The prepared MOS/COS/CC electrocatalyst showed excellent OER and HER activity in alkaline solution. In OER and HER tests, the MOS/COS/CC-6 h only required overpotentials of 197 and 140 mV at a current density of 10 mA/cm^2^ and exhibited lower Tafel slop. These excellent properties are attributed to the abundant active sites and the interaction of MoS_2_ and CoS_2_ nanosheets. The abundant active sites enable the material to exhibit excellent catalytic activity. Our proposed synthesis strategy can provide an effective tool for realizing high-performance electrocatalysis by exploiting of the synergy between individual nanostructures.

## Supplementary Information

** Additional file 1: Figure S1.** XPS spectrum of MoS/CoS/CC (a), (b) and (c) MoS/CoS/CC-4H, (d), (e) and (f) MOS/COS/CC-8 h. **Figure S2.** Mo 3d XPS spectrum of MOS/COS/CC-6 h and MoS_2_ electrocatalysts.
