# Supplementary material for: N-doped carbon anchored CoS2/MoS2 nanosheets as efficient electrocatalysts for overall water splitting
Source: Front Optoelectron. 2022 Jul 18;15(1):30. doi: 10.1007/s12200-022-00034-3 (PMC9756241; doi:10.1007/s12200-022-00034-3)
Supplement: Supplementary file 1 — Additional file 1: Figure S1. XPS spectrum of MoS/CoS/CC (a), (b) and (c) MoS/CoS/CC-4H, (d), (e) and (f) MOS/COS/CC-8 h. Figure S2. Mo 3d XPS spectrum of MOS/COS/CC-6 h and MoS2 electrocatalysts. [file 12200_2022_34_MOESM1_ESM.pdf]

Supporting Information

N-doped carbon anchored CoS_2/MoS_2 nanosheets as efficient electrocatalysts for overall water splitting

Xingwei Zhou[a], Wei Zhang[a,b,*], Zunhao Zhang[a], Zizhun Wang[a], Xu Zou[a,*], Dabing Li[c], Weitao Zheng[a]

[a] Key Laboratory of Automobile Materials MOE, School of Materials Science & Engineering, Jilin Provincial International Cooperation Key Laboratory of High-Efficiency Clean Energy Materials, Electron Microscopy Center, and International Center of Future Science, Jilin University, Changchun 130012, China

[b] Wuhan National Laboratory for Optoelectronics, Huazhong University of Science and Technology, Wuhan 430074, China

[c] State Key Laboratory of Luminescence and Applications, Changchun Institute of Optics, Fine Mechanics and Physics, Chinese Academy of Sciences, Changchun 130033, China

* Email: weizhang@jlu.edu.cn ; zoux@jlu.edu.cn

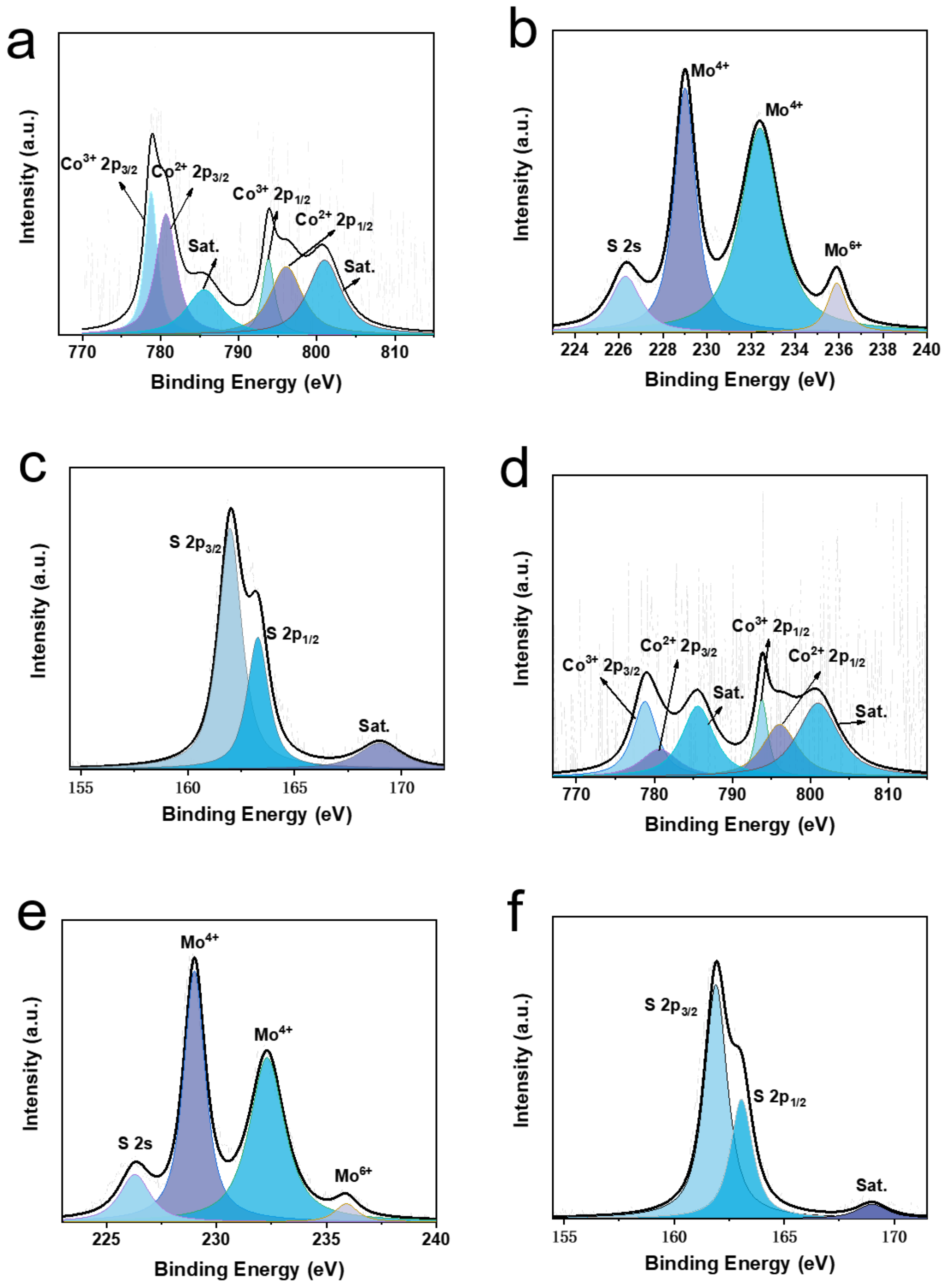

Figure S1 XPS spectrum of (a), (b) and (c) MoS/CoS/CC-4H, (d), (e) and (f) MOS/COS/CC-8H.

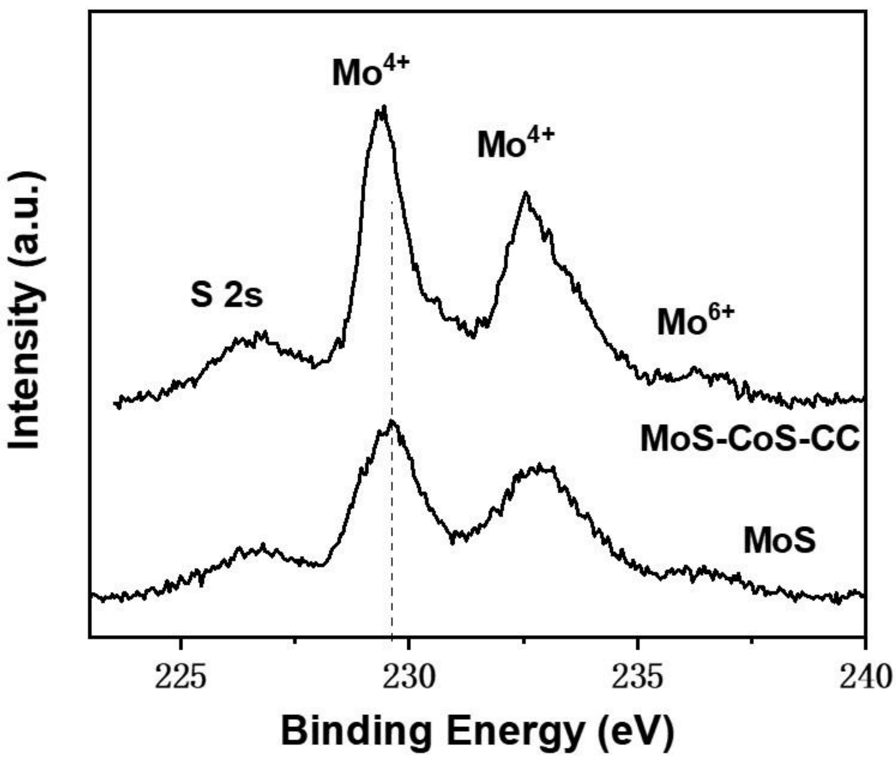

Figure S2 Mo 3d XPS spectrum of MOS/COS/CC-6H and MoS_2 electrocatalysts.
